# Prevalence of Anxiety in Dental Students during the COVID-19 Outbreak: A Meta-Analysis

**DOI:** 10.3390/ijerph182010978

**Published:** 2021-10-19

**Authors:** Javier Santabarbara, Nahia Idoiaga, Naiara Ozamiz-Etxebarria, Juan Bueno-Notivol

**Affiliations:** 1 Department of Microbiology, Pediatrics, Radiology and Public Health, University of Zaragoza, C/Domingo Miral s/n, 50009 Zaragoza, Spain; jsantabarbara@unizar.es; 2 Centro de Investigación Biomédica en Red de Salud Mental (CIBERSAM), Ministry of Science and Innovation, 28029 Madrid, Spain; 3 Aragonese Institute of Health Sciences (IIS Aragón), 50009 Zaragoza, Spain; 4 Department of Developmental and Educational Psychology, University of the Basque Country UPV/EHU, 48940 Leioa, Spain; naiara.ozamiz@ehu.eus; 5 Psychiatry Service, Hospital Universitario Miguel Servet, 50009 Zaragoza, Spain; elecrijuan@hotmail.com

**Keywords:** anxiety, dental students, gender, countries, meta-analysis, COVID-19

## Abstract

Background: Since the onset of the COVID-19 pandemic, the psychological state of university students has been a cause for concern. In particular, odontology students have experienced symptoms of anxiety due to the closure of universities and the suspension of clinical training. Methods: Medline via PubMed was searched for studies on the prevalence of anxiety in dental undergraduates, published from 1 December 2019 to 1 August 2021. Results: A total of fifteen studies were included in this review. Our results show a prevalence of anxiety of 35% reported by dental students, which was independent of gender, response rate or methodological quality. The only significant finding was a lower prevalence of anxiety in studies located in Europe compared to those located in other continents. Conclusions: The results suggest dental students are experiencing significant levels of anxiety during this COVID-19 pandemic and that there seem to be differences between students from different regions of the world. Therefore, it is important to help dental students psychologically as the pandemic situation continues.

## 1. Introduction

Since the WHO declared the global pandemic of COVID-19 in March 2020 [1], in addition to the medical consequences, the psychological and social impact that this pandemic involves is undisputed. As a result, studies on the psychological effects of the pandemic have been conducted worldwide in populations such as health professionals [2], the general population [3], the elderly [4], students [5,6], children [7], adolescents [8], caregivers [9] and teachers [10].

In this psychological impact, anxiety has been particularly prominent. The American Psychiatric Association describes anxiety as the anticipation of a future threat, accompanied by feelings of dysphoria or physical symptoms of tension [11]. In the same vein, anxiety has been defined as an unpleasant state of agitation and restlessness [12]. An emotional reaction to the perception of danger or threat manifests itself through a set of responses grouped into three systems: Cognitive or subjective, physiological or bodily and motor, jointly or independently [13]. However, in anxiety, the psychic symptoms, the sensation of suffocation and imminent danger, are usually more present, since a startle reaction is presented [14].

Among the studied populations, college students are a major public health concern that has intensified during the COVID-19 pandemic [15]. In addition to their pre-pandemic concerns, such as pressure to succeed or academic performance [16], they now face fear, uncertainty, social distancing measures, compliance with new norms, over-information and ambiguous messages from the media and social networks [17,18] and the loss of socialization [19,20], essential at this age. In addition, university students have had to adapt to online classes, and the quality and logistics of internships have been modified, leading to psychological distress [21]. A meta-analysis conducted in 15 different countries showed a higher prevalence of psychological symptomatology among university students during the pandemic compared to before the pandemic [15].

As for dental students, they were already considered one of the most stressed students as a result of the academic pressure, competitiveness and workload [22,23]. Dental students have also suffered from the educational modifications that have occurred during the pandemic, such as the shift to online education to comply with social distancing measures and minimize disease transmission [24]. However, online education cannot fully address the challenges and requirements of dental education, placing dental students under additional stress.

In the clinical years of dental school, students often work in close proximity to patients and are exposed to high levels of aerosols, droplets and oral fluids. This implies an increased risk of exposure and viral transmission, making it a profession with a high risk of COVID-19 infection [25]. Thus, it has been observed that dental professionals and dental students are the individuals most vulnerable to the risk of COVID-19 transmission [26]. Studies conducted with dental students show that they experience high levels of anxiety during the pandemic as they worry about infecting family and friends [27,28], but more so for those infected with coronavirus [24].

Among the risk factors, the female gender has been associated with worse mental health during the pandemic in different studies carried out in the general population [28,29,30] and in healthcare professionals [31,32], but there are also studies suggesting that there have been no symptomatologic differences between men and women [10,33,34]. As for university students, different meta-analyses [15,35] have also shown a higher prevalence of anxiety in women than in men.

Regarding geographical differences, studies in the general population [33] and in healthcare workers [32] show a similar prevalence of anxiety across countries and continents. However, a meta-analysis conducted in 15 countries focusing on university students found a lower prevalence in Asian countries [15].

Therefore, the present meta-analysis aims to update the evidence on the prevalence of anxiety in dental students during the pandemic. More specifically, we aim to analyze whether there are differences in the prevalence of anxiety according to gender and country of study.

## 2. Methodology

This study was conducted in accordance with the PRISMA guidelines for reporting systematic reviews and meta-analyses [36] (Supplementary Table S1) following the methodology of previous works [6].

### 2.1. Search Strategy

Two researchers (JBN and JS) conducted a literature search for all cross-sectional studies reporting the prevalence of anxiety in dental students published from 1 December 2019 to 1 August 2021, using MEDLINE via the PubMed database. The search strategy is detailed in Table 1.

No language restrictions were enforced. References from selected articles were inspected to detect additional potential studies. Then we performed a manual search of the “grey literature” (e.g., medRxiv or Google Scholar) to detect other potentially eligible investigations. Any disagreement was resolved by consensus among the third and fourth researchers (NO-E and NI).

### 2.2. Selection Criteria

Studies were included if they (1) reported cross-sectional data on the prevalence of anxiety, or sufficient information to compute this, conducted during the COVID-19 outbreak; (2) focused on dental students; (3) included a validated instrument to assess or diagnose anxiety; and (4) made the full text available.

We excluded studies focusing only on community-based samples of the general population or specific samples that were not dental students (e.g., medical students, medical professionals, patients), as well as review articles.

A pre-designed data extraction form was used to extract the following information: Country, sample size, prevalent rates of anxiety, the proportion of women, average age, instruments used to assess anxiety, response rate and sampling methods.

### 2.3. Methodological Quality Assessment

Articles selected for retrieval were assessed by two independent reviewers (JBN and JS) for methodological validity before they were included in the review using the Joanna Briggs Institute (JBI) standardized critical appraisal instrument for prevalence studies [37], recommended for the assessment of cross-sectional studies [38]. Quality was evaluated according to nine criteria, each yielding a score of zero or one. One score was obtained for each criterion if the study was affirmative to the following questions: 1: Was the sample frame appropriate to address the target population? 2: Were study participants recruited in an appropriate way? 3: Was the sample size adequate? 4: Were the study subjects and setting described in detail? 5: Was data analysis conducted with sufficient coverage of the identified sample? 6: Were valid methods used for the identification of the condition? 7: Was the condition measured in a standard, reliable way for all participants? 8: Was there appropriate statistical analysis? 9: Was the response rate adequate, and if not, was the low response rate managed appropriately?

Any disagreements between the reviewers were resolved through discussions, or by further discussion with a third or fourth researcher (NO-E and NI).

### 2.4. Data Extraction and Statistical Analysis

Freeman and Tukey’s double arcsine transformation of prevalence to stabilize the variance was applied [39]. A generic inverse variance method with a random effect model was used [40], which is more appropriate than fixed-effect models in the presence of heterogeneity between studies [41]. The Hedges Q statistic was reported to check heterogeneity across studies, with statistical significance set at *p* < 0.10. The I2 statistic and a 95% confidence interval (95% CI) was also used to quantify heterogeneity [42]. Values between 25% and 50% were considered low, 50–75% were moderate and 75% or more were high [43]. Heterogeneity of effects between studies occurs when differences in results for the same exposure–disease association cannot be fully explained by sampling variation. Sources of heterogeneity can include differences in study design or in demographic characteristics. We performed meta-regression and subgroup analyses [44] to explore the sources of heterogeneity expected in meta-analyses of observational studies [45]. We conducted a sensitivity analysis to determine the influence of each individual study on the overall result by omitting studies one by one. Publication bias was determined through visual inspection of a funnel plot as well as Egger’s test [46] (*p* values < 0.05 indicate publication bias) since funnel plots were found to be an inaccurate method for assessing publication bias in meta-analyses of proportion studies [47].

Statistical analyses were conducted by JS and run with STATA statistical software (version 10.0; College Station, TX, USA) and R [48].

## 3. Results

**Figure 1:**
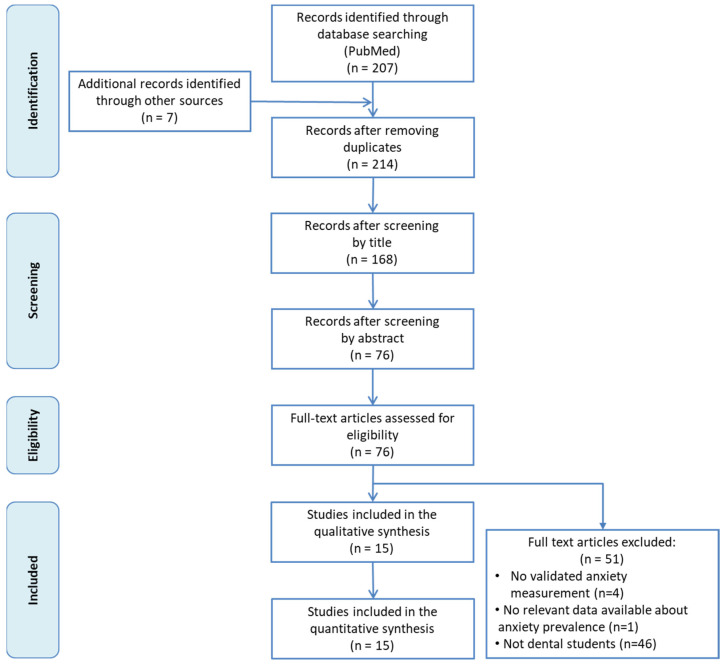
Flowchart of the study selection.

Figure 1 shows the flowchart of the literature search strategy and study selection process. In total, 207 records were initially identified from Medline via PubMed and 7 extra records were then added after a manual search in a preprints database (MedRxiv) and Google Scholar, from which 138 were excluded after a first screening of the titles and abstracts. After reading the remaining 76 articles in full, we finally included 15 in our meta-analysis [49,50,51,52,53,54,55,56,57,58,59,60,61,62,63]. Reasons for exclusion are detailed in Figure 1.

A description of the included studies is reported in Table 2. Most of the studies were carried out in Asia (n = 9), but we also found studies from Europe (n = 2) and North (n = 2) and South America (n = 2), with sample sizes ranging from 97 to 1050 participants. Most of the studies involved young students, and provided data referring to the academic year, while few articles reported a mean age among participants. Among the six articles that did, the mean age ranged from 21.31 to 23.45 years. All studies included both men and women, with a clear predominance of women in all studies. All studies were conducted using online questionnaires distributed either by email or through social media. Of those reporting the sampling methodology, all except two used non-random methods. The response rate was reported by nine studies and ranged from an estimation of 20% to 95.10%. All studies measured anxiety using standardized scales, most commonly the Generalized Anxiety Disorder scale (GAD-7, n = 7 studies) and the Depression, Anxiety and Stress Scale (DASS, n = 7 studies), with one study using the Zung Self-Rating Anxiety Scale (SAS).

The risk of bias scores ranged from 4 to 9 out of a possible total of 9, with a mean score of 6.7 (SD = 1.3) (Supplementary Table S2). The most common limitations were (a) the recruitment of participants was not appropriate (13 studies), (b) the response rate was not reported, or there was a large number of non-responders (9 studies) and (c) the sample size was too small to ensure good precision of the final estimate (7 studies).

**Figure 2:**
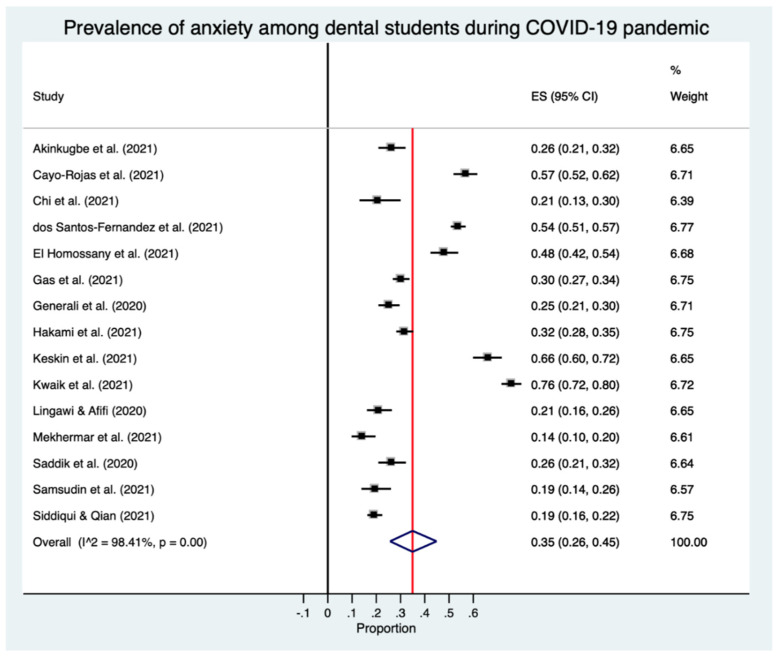
Forest plot for the prevalence of anxiety in dental students.

The estimated overall prevalence of anxiety was 35% in dental students (95% CI: 26–45%), with significant heterogeneity between studies (Q test: *p* < 0.001; *I*^2^ = 98.4%) (Figure 2).

Our meta-regression showed the prevalence of anxiety was independent of the percentage of women (*p* = 0.570), mean age at baseline (*p* = 0.410), response rate (*p* = 0.872) or methodological quality (*p* = 0.559). Neither sampling method was a significant moderator according to our subgroup analysis (*p* = 0.936). The only significant finding was a lower prevalence of anxiety for studies located in Europe (21% [95% CI: 18–24%]) compared to those located in other continents (America: 39% [95% CI: 24–55%]; Asia: 37% [95% CI: 24–51%]). We also observed a lower prevalence of anxiety for studies using the GAD-7 (31% [95% CI: 20–43%]) or the DASS-21 (36% [95% CI: 20–53%]) compared to those using the SAS (57% [95% CI: 52–62%]), although only one study [50] used the latter.

Excluding each study one-by-one from the analysis did not substantially change the pooled prevalence of anxiety, which varied between 32% (95% CI: 24–41%), with Kwaik et al. [63] excluded, and 37% (95% CI: 27–47%), with Mekhermar et al. [54] excluded. This indicates that no single study had a disproportional impact on the overall prevalence.

**Figure 3:**
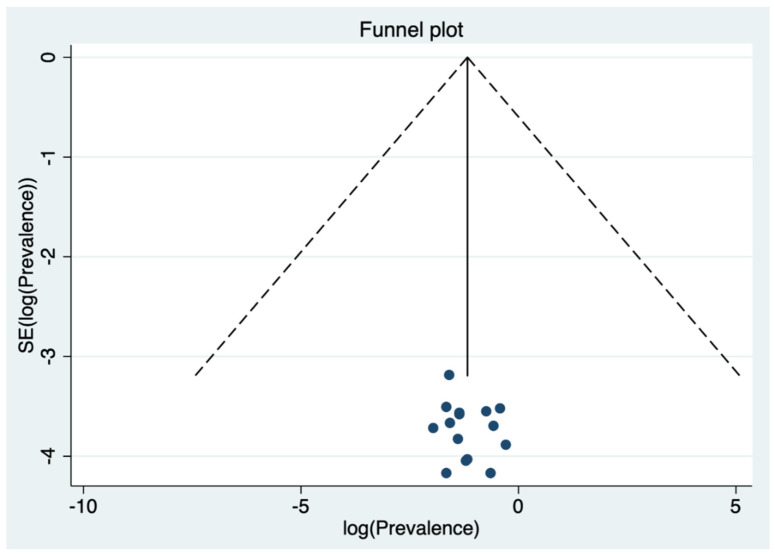
Funnel plot for the prevalence of anxiety in dental students.

Visual inspection of the funnel plot (Figure 3) suggested no presence of publication bias for the estimate of the prevalence of anxiety in dental students, confirmed by non-significant Egger test results (*p* = 0.529).

## 4. Discussion

### 4.1. Summary of Main Findings

Anxiety emerges as one of the most notable psychological consequences of the COVID-19 pandemic. University students in general, and dental students in particular, are one of the populations that are suffering the most from this symptomatology. In the present study, we have conducted a meta-analysis reporting on the prevalence of anxiety in dental students during the COVID-19 pandemic. It is based on 15 studies and, to our knowledge, it is the first meta-analysis that specifically reviews anxiety in dental students during the pandemic. The results of the present study show an estimated overall prevalence of anxiety in dental students of 35%. Although significant heterogeneity was found among the included studies, individually, none had a disproportional impact on the overall prevalence estimate.

Several systematic reviews and meta-analyses have examined the prevalence of anxiety during the Covid-19 crisis in overall university students. For instance, Batra el al. [15] in their meta-analysis of studies published up to July 2020 found a prevalence of anxiety in university students of 39.4%. The meta-analysis by Wang et al. [64] of studies published up to September 2020 found a prevalence of 31%, Li el al. [65] in their review up to October 2020 found a prevalence of 36%, Chang et al. [5] up to November 2020 found a prevalence of 31% and Deng et al. [35] found a prevalence of 32% up to January 2021. Furthermore, in a meta-analysis involving medical students conducted in August 2020, Lasheras et al. [6] found an incidence of anxiety of 28%. All these rates are higher than the 25% prevalence rate found by Santabarbara et al. [66] for the general population up to August 2020. Therefore, the present meta-analysis is consistent with earlier studies that reported that college students face higher levels of anxiety than the general population during the Covid-19 pandemic [67]. In addition, this review also points out that the anxiety prevalence among dental students is very similar to that among university students in general, although somewhat higher than that among medical students, something that had also been found in studies prior to the pandemic [68]. This symptomatology could have a negative impact on academic performance and, in the case of dental students, on professionalism and empathy towards patients during their training, as anxiety negatively influences interpersonal communication and empathy [69]. Anxiety has also been found to directly affect trainees’ confidence, as well as their clinical training [70]. Furthermore, empathy and communication are known to be very important skills in the practice of dental professionals, since, in addition to improving their care [71], they reduce patient anxiety and improve the negotiation of treatment plans, as well as adherence to them, thus increasing patient satisfaction [72].

In addition, beyond the academic-professional sphere, anxiety must also be taken into account on a personal level, as it is associated with a lower quality of life [73], a loss of interpersonal relationships [74], self-confidence [75] and higher rates of depression [76], among others. Moreover, the anxious and depressive state of college students can also influence their future studies and professionalism as practitioners [77].

It is also interesting to examine the prevalence of anxiety by subgroups. In this study, no significant differences were found among dental students when subdivided by gender. These results do not match with other findings in the general population, which indicate that women face more anxiety than men [54,66]. However, many meta-analyses conducted during the pandemic about university students have not found gender differences either [5,6,30,65]. This may be due to the characteristics of university students, usually young and without family responsibilities, which may have a stronger impact on women in other cohorts [78,79]. On the other hand, pre-pandemic studies found a significant increase in stress according to the dental students’ year of study [80]. In particular, students in higher grades tended to have more stress and anxiety when moving from preclinical to clinical training [81,82,83]. However, in this study, no differences in the prevalence of anxiety by age were found. This could be influenced by the fact that many of the clinical practices have stopped during the pandemic [84], so the difference in anxiety they might create has disappeared. In fact, studies have also found that some students have preferred to postpone their internships until the end of the Covid-19 pandemic or at least until getting vaccinated [51].

However, the most significant difference in the prevalence of anxiety between groups in this research was found for geographical region. There is a lower prevalence of anxiety in studies located in Europe (21%) compared to those located in other continents (America, 39%; Asia, 37%). It should be noted that in previous meta-analyses, dealing with both the general population and university students, Asia was the region with the lowest prevalence of anxiety [6], as well as other symptoms such as depression [15]. This hints at an important influence of culture- or region-specific characteristics, but since there are no other studies that agree with these results, they could perhaps be explained by the characteristics of dental studies in particular. It should be noted that only two of the studies were carried out in Europe and that the curriculum of dental studies has a great variability between different European countries [85]. Therefore, it would be relevant to further replicate these studies in different European countries or to conduct cross-national studies.

Furthermore, an international study conducted by Perry et al. [86] in 2017 about simulation and curriculum design in dental studies found that, in America, they had a more traditional curriculum, which also tended to be shorter, but with a higher number of simulation hours. As for Asia, along with Oceania, they were found to be the regions with the highest ratio of alternative learning methods, such as haptic simulations, the Phantom Laboratory or any simulated teaching aids. Therefore, it could be speculated that in both regions, students noticed the loss of this cynical practice more and therefore their anxiety levels increased more. In the same line, recent studies found that a high number of undergraduate dental students reported psychological problems linked to the challenge of moving from a laboratory environment to a clinical setting. That is, the question of whether they really have sufficient skills or have achieved sufficient competencies during their studies to move to a clinical setting was one of the most important sources of anxiety even before the pandemic [87]. However, now, with clinical practice at a standstill, this challenging transition could produce even more anxiety, since, with online teaching, many undergraduates believe they are not obtaining the same competencies as with traditional teaching [88].

Therefore, it is important to emphasize the importance of maintaining clinical practices in the training of dental students, albeit with maximum safety measures. In fact, maintaining safety in dental clinics is something that has been of concern to dental professionals, so several innovative treatments and procedures have been proposed in the literature [89]. In fact, from the review of these proposals, Cianetti et al. [89] have proposed a specific protocol detailing the steps to be followed both at the time of remote contact with the patient and at different moments within the dental clinic (while waiting, in the dental chair room and after the patient’s visit). Regarding the quality of the research analyzed, the results of this study indicate that the analyzed research has good methodological quality. So, to make it even better, we suggest researchers follow the recommendations of the Joanna Briggs Institute (JBI) standardized critical appraisal instrument for prevalence studies [37], or any other recognized risk of bias scale of their choice, on study design. It should be noted that considering the quality of the included studies, appropriate recruitment of participants and adequate sample size should be pursued, although in times of pandemic, and especially in periods of confinement, this may require greater effort for researchers. In a similar way, many of the surveys did not report their response rate or their response rates were low. This may be due to the fact that research had to be conducted online to ensure the required social distancing, making it more difficult to obtain answers to questionnaires from respondents.

In addition, some of the online questionnaire tools do not offer the option of knowing how many people have received the form and how many of them have responded, and therefore of measuring the response rate. All this must be taken into account, as the uniqueness of the timing should not be an excuse to reduce the quality of the research. Still, with an average score of 6.7 out of 9, we can consider the quality of the studies included in this review to be high. Furthermore, our analyses also show that neither methodological quality nor response rate influenced our prevalence estimate.

### 4.2. Strengths and Limitations

One of the strengths of the present meta-analysis is the inclusion of a large body of literature. Moreover, Egger’s test has been used to perform a rigorous approach in order to identify publication bias. Finally, to our knowledge, this is the first meta-analysis conducted so far on the prevalence of anxiety symptomatology in dental students during the COVID-19 pandemic. However, some limitations have to be taken into account when interpreting our results. Firstly, it should be noted that after performing the systematic review and meta-analysis, one article was found that met the inclusion criteria for our review but was not listed in Pubmed [90]. Yet, a sensitivity analysis revealed that its inclusion would not change the results significantly, with an overall prevalence estimate of 34% (95% CI 26–43%). In addition, another paper was discarded due to a lack of access to the full text [91]. Another limitation is that most of the included studies are based on non-probability samples and cross-sectional data from different points in time during the pandemic. As the epidemiological situation of COVID-19 is constantly changing, more longitudinal studies are needed to determine changes in anxiety levels over time [92]. Finally, the included studies have included a variety of self-report scales, yet it would be better if the studies used the same standardized measure of anxiety. Finally, it should be noted that, with the scales that were used in the studies, we have assessed students’ perceptions of their own anxiety, instead of a measure of the presence or absence of anxiety based on a clinical interview with a trained mental health professional.

## 5. Conclusions

Anxiety levels among dental students have previously concerned the scientific community [87], and such worries have persisted in pandemic times. This meta-analysis shows that the prevalence of anxiety among dental students during the COVID-19 pandemic is significant, being higher than the prevalence in the general population or in medical students.

Given that the pandemic is dragging on, there is a need to reach out to dental students, especially in those countries where the prevalence of anxiety has skyrocketed. It is also relevant to collect data in countries where it is currently not available in order to understand how national students are coping with the COVID-19 challenge. Indeed, strategies such as eHealth group interventions have already been recommended as they could significantly improve the psychological well-being of university students [93]. In fact, the lack of interventions and policies to improve mental health in universities can have psychological consequences later in their personal, academic and professional life [94]. Likewise, in the case of dental students, it will also be important to address how and with what health guarantees they are able to conduct their clinical practices, both directly with people and simulated, as this issue is key to the prevalence of anxiety.

## Figures and Tables

**Table 1:** Search strategy in Pubmed.

(covid[tiab] OR covid-19[tiab] OR coronavirus[tiab] OR SARSCoV-2[tiab] OR “Coronavirus”[Mesh] OR “severe acute respiratory syndrome coronavirus 2”[Supplementary Concept] OR “COVID-19”[Supplementary Concept] OR “Coronavirus Infections/epidemiology”[Mesh] OR “Coronavirus Infections/prevention and control”[Mesh] OR “Coronavirus Infections/psychology”[Mesh] OR “Coronavirus Infections/statistics and numerical data”[Mesh]) AND (anxiety OR anxiety symptoms OR anxiety disorders OR anxious OR “Trauma and Stressor Related Disorders”[Mesh] OR “Anxiety”[Mesh] OR “Anxiety Disorders”[Mesh] OR “Anxiety/epidemiology”[Mesh] OR “Anxiety/statistics and numerical data”[Mesh]) AND (“Students, Dental”[Mesh] OR “dental students”[tiab] OR “dental undergraduates”[tiab] OR “university students” [tiab])

**Table 2:** Description of studies included in the meta-analysis.

Author (Publication Year)	Country	Mean Age (SD)	% Females (n)	Sample Size (n)	Response Rate (%)	Sampling Method	Anxiety Assessment	Diagnostic Criteria	Prevalence		Quality Assessment
									%	n	
Akinkugbe et al. (2021)	USA	NR	62.30% (157)	252	58%	Convenience sampling	GAD-7	≥10	26.19%	66	7
Cayo-Rojas et al. (2021)	Peru	23.19 (4.2)	75.43% (304)	403	NR	Convenience sampling	SAS	≥45	56.82%	229	7
Chi el al. (2021)	USA	NR	52.58% (51)	97	35.5%	Convenience sampling	GAD-7	≥10	20.62%	20	6
dos Santos-Fernandez et al. (2021)	Brazil	23.27 (4.7)	70.38% (739)	1050	NR	NR	GAD-7	≥10	53.81%	565	7
El Homossany et al. (2021)	Saudi Arabia	NR	54.55% (168)	308	88.0%	Convenience sampling	GAD-7	≥10	48.05%	148	7
Gaș et al. (2021)	Turkey	21.31 (1.9)	64.66% (452)	699	95.1%	Random sampling	DASS-21	≥10	30.19%	211	9
Generali et al. (2020)	Italy	23.45 (3.3)	58.89% (227)	399	75%	Convenience sampling	GAD-7	≥10	25.06%	100	8
Hakami et al. (2021)	Saudi Arabia	21.76 (1.9)	54.82% (381)	695	NR	Cluster sampling	DASS-21	≥10	31.65%	220	8
Keskin (2021)	Turkey	NR	60.23% (156)	259	NR	Convenience sampling	DASS-42	≥10	66.02%	171	5
Kwaik et al. (2021)	Palestine	NR	81.19% (354)	436	55.18%	NR	DASS-21	≥10	75.92%	331	8
Lingawi & Afifi (2020)	Saudi Arabia	NR	59.69% (154)	258	86%	NR	GAD-7	≥10	20.93%	54	6
Mekhemar et al. (2021)	Germany	NR	73.46% (155)	211	NR	Convenience sampling	DASS-21	≥6	14.22%	30	5
Saddik et al. (2020)	UAE	NR	NR	244	NR	Convenience sampling	GAD-7	≥10	26.23%	64	4
Samsudin et al. (2021)	Malaysia	NR	79.43% (139)	175	94.6%	Convenience sampling	DASS-21	≥10	19.43%	34	6
Siddiqui & Qian (2021)	Malaysia	22.45 (NR)	79.24% (519)	655	20%	Convenience sampling	DASS-21	≥10	19.24%	126	7

Note. Quality score based on the Joanna Briggs Institute (JBI) standardized critical appraisal instrument for prevalence studies [37]. DASS-21 = Depression, Anxiety and Stress scale; GAD-7 = Generalized Anxiety Disorder scale; NR = not reported; SAS = Zung Self-Rating Anxiety Scale; UAE = United Arab Emirates; USA = United States of America.

## Data Availability

Not applicable.

## Supplementary Materials

The following are available online at https://www.mdpi.com/article/10.3390/ijerph182010978/s1, Table S1: PRISMA Checklist, Table S2: Quality assessment.
