# Supplementary material for: Prevalence of Anxiety in Dental Students during the COVID-19 Outbreak: A Meta-Analysis
Source: Int J Environ Res Public Health. 2021 Oct 19;18(20):10978. doi: 10.3390/ijerph182010978 (PMC8535371; doi:10.3390/ijerph182010978)
Supplement: Supplementary file 1 [file ijerph-18-10978-s001.zip › Supplementary_Table_S2_revJS.pdf]

Supplementary Table S2. Quality assessment.

Study	1	2	3	4	5	6	7	8	9	TOTAL
Akinkugbe *et al.* (2021)	Y	N	N	Y	Y	Y	Y	Y	Y	7
Cayo-Rojas *et al.* (2021)	Y	N	Y	Y	Y	Y	Y	Y	U	7
Chi *el al.* (2021)	Y	N	N	Y	Y	Y	Y	Y	N	6
dos Santos-Fernandez *et al. (2021)*	Y	U	Y	Y	Y	Y	Y	Y	U	7
El Homossany *et al.* (2021)	N	N	Y	Y	Y	Y	Y	Y	Y	7
Gaş *et al.* (2021)	Y	Y	Y	Y	Y	Y	Y	Y	Y	9
Generali *et al.* (2020)	Y	N	Y	Y	Y	Y	Y	Y	Y	8
Hakami *et al.* (2021)	Y	Y	Y	Y	Y	Y	Y	Y	U	8
Keskin (2021)	N	N	N	Y	Y	Y	Y	Y	U	5
Kwaik *et al.* (2021)	Y	U	Y	Y	Y	Y	Y	Y	Y	8
Lingawi & Afifi (2020)	Y	U	N	Y	Y	Y	Y	Y	U	6
Mekhemar *et al.* (2021)	Y	N	N	N	Y	Y	Y	Y	U	5
Saddik *et al.* (2020)	U	N	N	N	Y	Y	Y	Y	U	4
Samsudin *et al.* (2021)	Y	N	N	N	Y	Y	Y	Y	Y	6
Siddiqui & Qian (2021)	Y	N	Y	Y	Y	Y	Y	Y	N	7

Abbreviations: N: No, Y: Yes, U: Unclear; 1: Was the sample frame appropriate to address the target population?; 2: Were study participants recruited in an appropriate way?; 3: Was the sample size adequate?; 4: Were the study subjects and setting described in detail?; 5: Was data analysis conducted with sufficient coverage of the identified sample?; 6: Were valid methods used for the identification of the condition?; 7: Was the condition measured in a standard, reliable way for all participants?; 8: Was there appropriate statistical analysis?; 9: Was the response rate adequate, and if not, was the low response rate managed appropriately?
